# The Experiences of Clinicians Conducting Neurodiversity-Affirmative Formal Autism Identification (Assessment) for Children and Young People: A Phenomenological Study

**DOI:** 10.1192/bjo.2026.11230

**Published:** 2026-06-30

**Authors:** Evelyn Crosskey

**Affiliations:** Brighton and Sussex Medical School, Brighton, United Kingdom

## Abstract

**Aims:**

The clinical presentation of autism has been summarised in the 5th edition of the American Psychological Association’s Diagnostic and Statistical Manual for Mental Disorders (DSM–5). Assessment tools such as the Autism Diagnostic Observation Schedule have been developed to provide clinicians with the practical means to diagnose autism according to DSM–5. These medicalised assessments are deficit-based, focussing on what the autistic person cannot do compared to a non-autistic person. This juxtaposition can adversely affect the mental health of the autistic person and those who support them. The assessment can also place emotional burden upon the clinicians conducting them. The wider implication of the negative framing used in traditional tools is to perpetuate the stigma surrounding autism. Neurodiversity-affirmative identification is a new approach for clinicians. This methodology reframes the tragedy narrative of Autism to offer clinicians the means to work in collaboration with their clients to explore their autistic identity and not only identify their specific support needs but also their strengths. An understanding and acceptance of one’s sense of self world facilitates nurturing good mental health and improved life trajectories. This novel study examined the effect of the neurodiversity-affirmative way of working upon clinicians and how they make sense of the experience.

**Methods:**

The experiential qualitative methodology of interpretative phenomenological approach was used. Phenomenology is relevant as it is the philosophical study of what it is like to be human in our lived experiences and what matters to the individual. Secondly, and more specifically, interpretative phenomenology is appropriate as it is more than merely gathering a description, but an analysis of the participants’ attempts to interpret their personal and subjective lived experiences. Semi-structured, qualitative interviews were conducted with clinicians who are practicing neurodiversity-affirmatively between September and October 2025. The sample size of five clinicians complied with the usual approach for such qualitative projects.

**Results:**

Five Group Experiential Themes were identified: pressure to accept traditional framing of Autism, haunted by the past, wrong assumptions, more than a job, it’s a vocation and validating Autistic experience.

**Conclusion:**

The impact of conducting medical model assessments has been long-lasting and traumatic upon the clinicians. The results charted their intellectual transitioning from passive acceptance of the traditional assessment status quo to actively questioning it and finally being resolute that it needs to be revised and reframed and that the neurodiversity-affirmative approach needs to be widely adopted to better validate the autistic people they serve.

